# Mechanisms of AMPA Receptor Endosomal Sorting

**DOI:** 10.3389/fnmol.2018.00440

**Published:** 2018-12-05

**Authors:** Gabrielle T. Parkinson, Jonathan G. Hanley

**Affiliations:** Centre for Synaptic Plasticity and School of Biochemistry, University of Bristol, Bristol, United Kingdom

**Keywords:** endosome, synapse, trafficking, glutamate receptor, LTD (long term depression), LTP (long term potentiation), synaptic plasticity

## Abstract

The regulation of synaptic AMPA receptors (AMPARs) is critical for excitatory synaptic transmission, synaptic plasticity and the consequent formation of neural circuits during brain development and their modification during learning and memory processes. The number of synaptic AMPARs is regulated through endocytosis, exocytosis and endosomal sorting that results in recycling back to the plasma membrane or degradation in the lysosome. Hence, endo-lysosomal sorting is vitally important in maintaining AMPAR expression at the synapse, and the dynamic regulation of these trafficking events is a key component of synaptic plasticity. A reduction in synaptic strength such as in long-term depression (LTD) involves AMPAR sorting to lysosomes to reduce synaptic AMPAR number, whereas long-term potentiation (LTP) involves an increase in AMPAR recycling to increase the number of AMPARs at synapses. Here, we review our current understanding of the endosomal trafficking routes taken by AMPARs, and the mechanisms involved in AMPAR endosomal sorting, focussing on the numerous AMPAR associated proteins that have been implicated in this complex process. We also discuss how these events are dysregulated in brain disorders.

## Introduction

AMPA receptors (AMPARs) are ionotropic glutamate receptors that comprise hetero-tetrameric assemblies of subunits GluA1–4. Since AMPARs facilitate the majority of fast excitatory neurotransmission in the brain, changes in their abundance at synapses can significantly strengthen or weaken synaptic transmission (Malinow and Malenka, 2002). Long-term synaptic plasticity is thought to be a molecular and cellular correlate of learning and memory by playing a critical role in experience-dependent tuning of neural circuits that encode memories or behaviors (Mayford et al., 2012; Takeuchi et al., 2013). A decrease in synaptic strength involves a removal of AMPARs from synapses in long-term depression (LTD), whereas an increase in the number of synaptic AMPARs leads to increased synaptic strength known as long-term potentiation (LTP; Bredt and Nicholl, 2003; Henley and Wilkinson, 2016). In addition, homeostatic plasticity, also known as synaptic scaling, involves a cell-wide adjustment of synaptic strength to maintain a stable output of a particular neuron during changes in neuronal circuit activity (Fernandes and Carvalho, 2016).

**Figure 1:**
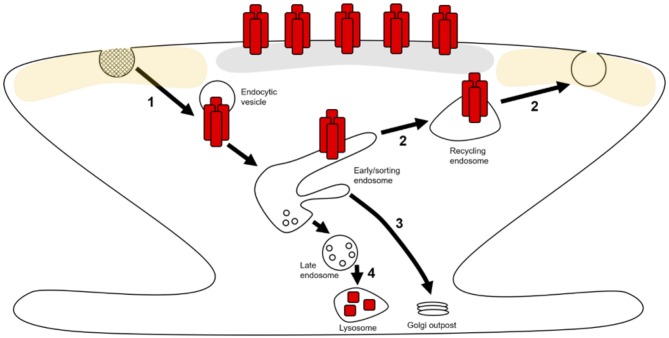
AMPA receptor (AMPAR) trafficking in dendritic spines and local dendrites. AMPARs (red rectangles) can laterally move along extrasynaptic (yellow shaded area) and dendritic plasma membranes where they can be captured and stabilized at the postsynaptic density (PSD; gray area). AMPARs can then be retrieved from the surface by endocytosis (1) and trafficked to early endosomes (EEs). At these EEs, receptors are sorted into distinct trafficking pathways. Receptors can recycle back to the plasma membrane through tubular domains on EEs via exocytosis at extrasynaptic zones (2). Through these tubular domains, AMPARs can also be retrogradely trafficked to Golgi outposts (3). The vesicular domains on EEs mature into late endosomes (LEs), which subsequently fuse with lysosomes resulting in receptor degradation (4).

Both basal maintenance and activity-dependent alterations of synaptic AMPAR expression are underpinned by the regulation of AMPAR trafficking through endosomal compartments within neurons (Hirling, 2009; Henley and Wilkinson, 2016). The constitutive and activity-dependent trafficking of AMPARs from the synaptic plasma membrane into intracellular compartments occurs predominantly through clathrin-mediated endocytosis (CME; Man et al., 2000; Cosker and Segal, 2014). Following internalization, cargo is trafficked to early endosomes (EEs) where it is sorted into distinct pathways. There are 3 possible routes that AMPARs can take from EEs: (1) a recycling path that returns cargo back to the plasma membrane via recycling endosomes (REs; Figure 1—step 2; van der Sluijs and Hoogenraad, 2011); (2) EEs can mature into late endosomes/multivesicular bodies (LEs/MVBs) and subsequently lysosomes to degrade the cargo contained therein (Figure 1—step 4; Hu et al., 2015); (3) cargo can be targeted from EEs back to the biosynthetic machinery for further post-translational modification (PTM; Figure 1—step 3; Hirling, 2009; van der Sluijs and Hoogenraad, 2011; Burd and Cullen, 2014).

It is estimated that approximately 60%–75% of all AMPARs in hippocampal neurons are intracellular (Richmond et al., 1996; Greger et al., 2003), and this internal pool contributes substantially to the regulation of surface AMPAR expression during constitutive and activity-dependent trafficking events. Indeed, an intracellular pool of AMPARs is proposed to function as a source of AMPARs for AMPAR synaptic delivery during LTP (Park et al., 2004; Kneussel and Hausrat, 2016) and forward AMPAR trafficking from these pools has been proposed to be negatively regulated during LTD (Lee et al., 2004; Citri et al., 2010).

AMPAR and NMDA receptor (NMDAR) stimulation have been shown to induce AMPAR trafficking to lysosomal compartments and their subsequent degradation (Ehlers, 2000; Lee et al., 2004) and blocking AMPAR lysosomal targeting inhibits hippocampal LTD (Fernández-Monreal et al., 2012). Thus, it is generally thought that increasing synaptic AMPARs during LTP-driven trafficking events requires the sorting of AMPARs into REs so that they return to the plasma membrane, whereas decreasing synaptic AMPARs during LTD plasticity events requires the retention of AMPARs at intracellular compartments and/or the active sorting of AMPARs towards LEs/lysosomes to be degraded.

These observations are complicated by the fact that individual AMPAR subunits confer different functional properties as well as different trafficking behaviors to the receptor complex (Bredt and Nicholl, 2003; Shepherd and Huganir, 2007; Henley and Wilkinson, 2016). Distinct trafficking mechanisms depend on the heterogeneity of AMPAR subunit C-terminal domains and the resultant diversity in interacting proteins (Passafaro et al., 2001; Lee et al., 2003; Anggono and Huganir, 2012). The majority of AMPARs are an assembly of two heterodimers of GluA2 and GluA1 or to a lesser extent GluA2 and GluA3 (Wenthold et al., 1996; Lu et al., 2009) and can only become incorporated into the synaptic membrane in tetrameric assemblies (Grunwald and Kaplan, 2003). The presence of GluA2 is of functional importance because it confers Ca^2+^ impermeability to the AMPAR channel (Isaac et al., 2007). GluA2 subunit largely determines constitutive and activity-dependent AMPAR endocytosis and recycling under resting conditions and sorting to lysosomes for degradation upon LTD induction. The presence of GluA3 subunit is also thought to promote lysosomal targeting (Lee et al., 2004). On the other hand, GluA1 subunits regulate LTP-induced AMPAR recycling (Hayashi et al., 2000; Shi et al., 2001; Park et al., 2004). These results are corroborated by the observation that the rate of basal GluA1 plasma membrane insertion is slow and enhanced by NMDAR activation, whereas GluA2-containing AMPAR exocytosis is faster under resting conditions and unaffected by NMDAR activity (Passafaro et al., 2001; Shi et al., 2001).

## Neuronal Endosomal Organization

It is generally thought that neurons utilize the same endosomal sorting system as non-neuronal cells (Figure 1). Nevertheless, due to their distinct morphology and cargo, neurons exhibit some unique aspects of endosomal compartment organization and regulatory trafficking mechanisms (Kennedy and Ehlers, 2006; Yap and Winckler, 2012).

Synaptic proteins, such as AMPARs, are concentrated and stabilized in dendritic spines in specialized structures called postsynaptic densities (PSDs) through interactions with scaffolding proteins. Prior to endocytosis, AMPARs must dissociate from the PSD scaffold, and are thought to move laterally to endocytic zones localized adjacent to the PSD (Ashby et al., 2004; Lu et al., 2007; Tao-Cheng et al., 2011; Opazo et al., 2012). The mechanisms of endocytosis *per se* will not be discussed in detail here. Once internalized, AMPARs enter EEs, which are short-lived endomembrane structures where cargo is sorted into distinct endosomal microdomains to be recycled, retrogradely trafficked or degraded (Scott et al., 2014). EEs in mammalian cells have morphologically distinct subdomains; tubular structures are thought to provide a greater surface area to volume ratio to capture the majority of membrane cargo for default recycling (Maxfield and McGraw, 2004; Collinet et al., 2010). Alternatively, some tubular domains are specialized to direct cargo back to the TGN (Burd and Cullen, 2014). In contrast, cargo marked for degradation are sorted into more bulbous regions that rapidly acidify and mature into LEs (Huotari and Helenius, 2011). Finally, LEs fuse with lysosomes, where hydrolases, proteases and lipases break down cargo in the intraluminal vesicles (Hu et al., 2015).

Neuronal EEs are found throughout the soma and dendrites, but are largely absent from axons (Ehlers, 2000; Wilson et al., 2000). In dendrites, approximately 70% of intracellular membrane structures are situated within or at the base of spines and 36%–56% of spines contain an endosomal structure, depending on developmental age (Cooney et al., 2002; Park et al., 2006; Kennedy et al., 2010). Large EEs can serve approximately 20 spines, although this number is smaller in the case of larger, more mature spines. REs and LEs are similarly localized throughout dendrites and the soma (Park et al., 2006; Von Bartheld and Altick, 2011). Indeed, it has been shown that the positioning of recycling endosomal compartments at the base of spines is crucial for synaptic AMPAR delivery and for spine morphology (Park et al., 2006; Esteves da Silva et al., 2015). Lysosomes were initially believed to be somatically restricted, but recent evidence has demonstrated that functional lysosomes are present in distal dendrites and can be recruited to spines during neuronal activity to degrade important cargo, including AMPARs (Goo et al., 2017; Padamsey et al., 2017). These specializations are thought to be necessary to quickly regulate endosomal trafficking events in the complex neuronal architecture (Hanus and Schuman, 2013).

## Detailed Molecular Mechanisms of AMPAR Endosomal Trafficking

**Figure 2:**
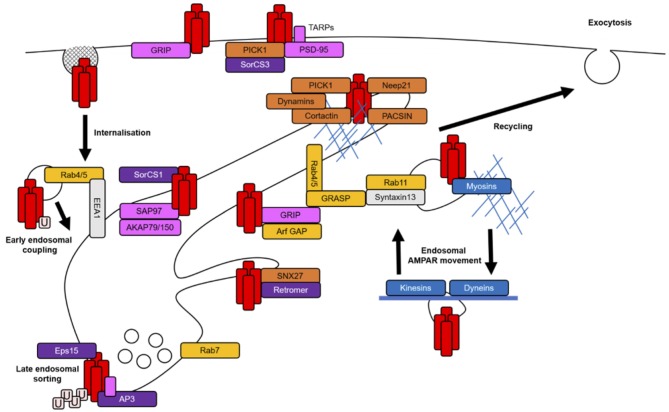
AMPAR-associated proteins in endosomal trafficking routes. AMPARs (red rectangles) are stabilized at the PSD by scaffolding proteins (pink rectangles). AMPARs are internalized largely via clathrin-mediated endocytosis (CME). AMPARs may be tagged for internalization by a single ubiquitin (pale pink squares) and those destined to be degraded are polyubiquitinated. The entry of AMPARs into the early endosomal system is mediated by the tethering protein EE antigen 1 (EEA1) and small GTPases and their effectors (yellow rectangles). The sorting of AMPARs at EEs involves protein complexes that link AMPARs to late or recycling endosomal compartments during coating and initial membrane budding events (purple rectangles). For example, adaptor protein 3 (AP3) and epidermal growth factor receptor substrate 15 (Eps15) in degradative paths and retromer and SorCS1 in recycling paths. The latter stages of AMPAR sorting also require the recruitment of proteins with diverse functions, such as PICK1, sorting nexin 27 (SNX27), protein kinase C and casein kinase II substrate in neurons (PACSIN) and Neep21, which complete the budding process via actin remodeling or generation of membrane curvature and prepare AMPARs for the next stages of their trafficking (orange rectangles). All stages of AMPAR sorting require small GTPase activation for the coordination of protein signaling and recruitment; Rab4/5 for endosomal entry, Rab7 for degradation and Rab11 or Arfs for recycling pathways. The actin cytoskeleton (thin blue lines) is mainly thought to facilitate endosomal transport in dendritic spines when coupled with certain associated proteins (blue rectangles), such as myosins. Microtubules (thick blue line) regulate longer range transport and promote the delivery of AMPARs to distal dendrites.

Due to the interconnected nature of the endomembrane system, it is likely that certain proteins function at multiple endosomal compartments to mediate the progression of one trafficking event to the next or indeed travel with cargo throughout intracellular membranes. Cargo-associated proteins involved in trafficking often contain various protein-binding domains, membrane-binding domains, GTPase regulatory domains or actin regulatory domains to mediate the complex interplay between membrane curvature, signaling cascades and the actin cytoskeleton that are necessary for coordinating membrane fission and fusion between endosomal compartments (Figure 2). The specific targeting of cargo from one membrane compartment to the next is determined by the interactions between these regulatory proteins and the cargo itself (Bonifacino and Glick, 2004).

### Endosomal Entry

For AMPARs to enter the endosomal system they must be released from their synaptic stabilizing proteins such as GRIP and PSD-95 (Bats et al., 2007). The majority of LTD-induced AMPAR internalization is believed to occur through CME (Man et al., 2000; Collingridge et al., 2010), and it has been suggested that constitutive internalization may occur via clathrin-independent endocytosis (Glebov et al., 2015; Fujii et al., 2017). There are a number of proteins that interact with AMPARs and the endocytic machinery for effective, activity-dependent, subunit-selective AMPAR internalization, the details of which are beyond the scope of this review (for a detailed review see Anggono and Huganir, 2012).

Specific mechanisms of AMPAR entry into the endosomal system after endocytosis are poorly understood, but the process requires Rab5 and EE antigen 1 (EEA1). EEA1 is a vesicle tethering protein that associates with phosphatidylinositol 3-phosphate (PI(3)P) in the EE membrane (Gaullier et al., 2000) and binds Rab5 on an endocytic vesicle to facilitate membrane fusion and hence incorporation of endocytic cargo into EEs (Simonsen et al., 1998; Murray et al., 2016). EEA1 inhibition or downregulation results in increased AMPAR conductance and GluA1-containing AMPAR surface expression (Selak et al., 2006; Xu and Pozzo-Miller, 2017). Rab5 overexpression results in increased EE to lysosome maturation, and increased lysosomal degradation of AMPARs (Lai et al., 2009). Consistent with these observations, Rab5 activity is essential for LTD (Brown et al., 2005), and the Rab5 GEF, RIN1, has recently been shown to facilitate activity-dependent AMPAR internalization (Szíber et al., 2017). However, it has not been definitively shown whether disrupting Rab5 function affects only AMPAR internalization or whether vesicle to endosome fusion is also interrupted.

### Endosomal Sorting

From EEs, a highly regulated process is needed to correctly sort AMPARs to the appropriate subsequent trafficking compartments. The sorting of cargo for degradation involves its localization to intraluminal MVB vesicles in the bulbous region of EEs (Babst, 2011). Subsequent sorting into LEs is mediated by ubiquitin interacting motif containing proteins, such as epsins, Hrs and Golgi-localized, gamma ear-containing Arf-binding proteins (GGAs) and the endosomal sorting complexes required for transport I, II, III (ESCRT I, II, III) machinery (Hicke and Dunn, 2003; Piper and Luzio, 2007; Babst, 2011). Until relatively recently, plasma membrane receptor recycling in mammalian cells was thought to be passive and rely heavily on the greater surface area of recycling tubular endosomal compartments to trap cargo for default return to the plasma membrane (Puthenveedu et al., 2010). However, many active sorting mechanisms into recycling pathways are being discovered.

### Post-translational Modifications

Dynamic PTMs such as phosphorylation, palmitoylation and ubiquitination influence AMPAR trafficking via numerous and varied mechanisms (Lu and Roche, 2012). For example, NMDAR-dependent AMPAR internalization during LTD involves GluA1 dephosphorylation at S845 (Lee et al., 1998; Ehlers, 2000), whereas AMPAR recycling during LTP involves GluA1 phosphorylation at S845 and S831 (Oh et al., 2006; He et al., 2009; Lee et al., 2010). On the other hand, phosphorylation of GluA2 subunit is regulated mainly in response to LTD induction to trigger AMPAR internalization (Kim et al., 2001; Ahmadian et al., 2004). While these signaling events have primarily been attributed to altering endocytosis rates, GluA1 S845 phosphonull mutants have been shown to degrade more rapidly in lysosomal compartments, instead of recycling back to the plasma membrane (He et al., 2009).

The most studied PTM involved in AMPAR sorting to degradative pathways is ubiquitination, which is the covalent addition of a 76 amino acid ubiquitin tag to lysine residues of a targeted protein. Although it is unclear whether AMPAR ubiquitination occurs at the postsynaptic plasma membrane (Patrick et al., 2003; Schwarz et al., 2010), or in EEs (Lussier et al., 2011; Widagdo et al., 2015), these ubiquitination events represent an early sorting event that can target cargo for degradation. All 4 AMPAR subunits can be ubiquitinated; GluA1 at K868 and GluA2 at K870 and K882 (Schwarz et al., 2010; Lussier et al., 2011; Widagdo et al., 2015). Lysine to arginine “ubiquitin-null” mutations at these sites result in reduced degradation and lysosomal targeting of AMPAR subunits (Lin et al., 2011; Widagdo et al., 2015). Moreover, expression of ubiquitin mutants that lack the ability to form polyubiquitin chains also prevent AMPAR internalization (Patrick et al., 2003). However, ubiquitination does not appear to be downstream of NMDAR activation, and so is unlikely to account for AMPAR degradation during LTD (Lussier et al., 2011; Widagdo et al., 2015). E3 ubiquitin ligases are critical for conjugating ubiquitin to substrates, and Nedd4-1 is recruited to synapses to increase ubiquitin-mediated AMPAR degradation following long-term bicuculline treatments (>20 h) to induce homeostatic down-scaling. This homeostatic mechanism is directly antagonized by NMDAR-dependent activation of the deubiquitinating enzyme ubiquitin carboxyl-terminal hydrolase 8 (USP8), to favor AMPAR deubiquitination and therefore AMPAR recycling (Scudder et al., 2014). RNF1 is another E3 ligase that is found at neuronal plasma membranes where it ubiquitinates AMPARs in response to AMPAR stimulation to decrease their surface expression via lysosomal degradation (Lussier et al., 2012).

Mechanistically, PTMs select AMPARs for recycling or degradation by altering their interactions with accessory trafficking proteins, which are discussed in the following sections. However, in the case of some PTMs, relevant regulated protein-protein interactions have not been identified.

### Scaffolding Proteins

Scaffolding proteins contain multiple interaction domains to bring important signaling or trafficking proteins into close proximity to AMPARs. GRIP contains seven PDZ domains that can simultaneously bind different PDZ ligand-containing proteins and maintains AMPAR surface expression via direct interaction with GluA2/3. The precise mechanism is still a matter of debate, and early studies hypothesized that the GRIP-AMPAR interaction functioned to stabilize AMPARs at the synaptic membrane (Dong et al., 1999; Osten et al., 2000), but it was later suggested that GRIP links AMPAR-containing REs to kinesin motor proteins and exocytic proteins to facilitate endosomal recycling back to the plasma membrane (Setou et al., 2002; Mao et al., 2010; Thomas et al., 2012). The GRIP-AMPAR interaction is blocked by phosphorylation of GluA2 at Ser880, which is regulated in response to LTD induction (Chung et al., 2000; Kim et al., 2001; Chung et al., 2003) or synaptic scaling (Tan et al., 2015). Additional mechanistic insight into GRIP function is provided by the observation that NSG1/Neep21, a small transmembrane protein that regulates the recycling of AMPARs in basal neuronal conditions interacts with GRIP. This interaction selectively promotes the return of GluA2-containing AMPARs to the plasma membrane, thereby diverting them from lysosomal degradation (Alberi et al., 2005; Steiner et al., 2005).

SAP97 is a PDZ domain containing scaffolding protein and a member of the membrane-associated guanylate kinase (MAGUK) protein family, which binds directly to GluA1 subunit and is thought to be involved in delivering AMPARs to the plasma membrane from intracellular compartments (Leonard et al., 1998; Schlüter et al., 2006). SAP97 forms a complex with GluA1-containing AMPARs and the actin motor protein myosin VI, which supports a role for SAP97 in AMPAR endocytosis and recycling (Wu et al., 2002; Osterweil et al., 2005; Nash et al., 2010). Although myosin VI is usually associated with cargo transport away from the plasma membrane towards actin minus ends, it should be noted that actin polarity is not uniform in mature dendritic spines and so myosin VI could regulate AMPAR traffic towards or away from the plasma membrane in dendritic spines (Nash et al., 2010). However, controversy remains about whether SAP97 regulates basal and/or activity-dependent AMPAR trafficking during LTP (Nakagawa et al., 2004; Schlüter et al., 2006; Howard et al., 2010). These discrepancies are likely due to the incomplete understanding of SAP97 splice variants in early studies and the functional redundancy within the MAGUK protein family (Howard et al., 2010; Liu et al., 2014).

Membrane-associated guanylate kinase inverted 2 (MAGI2) is structurally related to MAGUKs in that it contains a guanylate kinase-like domain and multiple PDZ domains, and proposed to act as an AMPAR scaffolding protein. MAGI2 interacts with AMPARs via TARPs (Deng et al., 2006), and is thought to maintain an intracellular pool of AMPARs, and hence constitutive AMPAR recycling to the plasma membrane (Danielson et al., 2012a,b).

AKAP79/150 interacts with AMPARs indirectly via SAP97 and contains binding domains for several kinases and phosphatases, such as PKA and calcineurin (Sanderson and Dell’Acqua, 2011). It is essential for anchoring PKA and calcineurin in close proximity to homomeric GluA1 AMPARs, so that they are transiently recruited to the synapse during LTD, LTP and homeostatic plasticity (Lu et al., 2007; Sanderson et al., 2018). AKAP79/150 is a substrate for the palmitoyl acyl transferase DHHC2, which is associated with REs (Greaves and Chamberlain, 2011; Woolfrey et al., 2015). Palmitoylation of AKAP79/150 by DHHC2 is required for AMPAR trafficking to the plasma membrane during LTP, probably as a result of more efficient PKA-dependent GluA1 phosphorylation (Keith et al., 2012; Woolfrey et al., 2015). It has also been suggested that CaMKII may cause the depalmitoylation of AKAP79/150 by an unknown mechanism, resulting in its removal from endosomal membranes in spines during LTD (Woolfrey et al., 2018). This may also influence AMPAR trafficking, but has not been empirically tested.

In summary, AMPAR scaffolding proteins contain a variety of protein interaction domains to coordinate complex trafficking processes by clustering AMPARs, auxiliary subunits, kinases and phosphatases onto intracellular membrane compartments and the plasma membrane. The complement of proteins recruited by scaffolding proteins and the consequent signaling events influence how and when the next stage of the trafficking process takes place.

### Auxiliary AMPAR Subunits

The core pore-forming AMPAR subunits associate with several families of transmembrane proteins, often referred to as auxiliary subunits, which alter AMPAR channel properties and trafficking (Sumioka, 2013; Haering et al., 2014; Jacobi and von Engelhardt, 2018). The most studied family of auxiliary subunits are the TARPs, originally identified as calcium channel γ subunits (Jackson and Nicoll, 2011; Greger et al., 2017). The prototypical TARP stargazin/γ2 interacts directly with AMPAR subunits to maintain their surface expression and facilitate clustering via PSD95 interactions (Chen et al., 2000; Schnell et al., 2002; Tomita et al., 2005; Bats et al., 2007). Additional AMPAR auxiliary subunits have been identified, for example cysteine-knot AMPAR modulating proteins (CKAMPs), which also influence AMPAR channel activity and regulate AMPAR trafficking through the biosynthetic pathway (Schwenk et al., 2012; Farrow et al., 2015). Since cargo exit from all intracellular membrane compartments are mechanistically similar and AMPAR auxiliary subunits interact with a variety of endosomal trafficking proteins, it is possible that similar mechanisms regulate AMPAR exit from biosynthetic and endosomal compartments. Indeed, TARPγ2 has been shown to interact with the adaptor protein (AP) complex AP-3 to promote AMPAR late endosomal/lysosomal trafficking (Matsuda et al., 2013). Moreover, another AMPAR interacting protein, PICK1, which regulates AMPAR trafficking at secretory and endosomal membranes (Lu et al., 2014; Mignogna et al., 2015), forms a complex with protein kinase C alpha (PKCα) and CKAMP44 (Kunde et al., 2017). Thus, it will be interesting to determine how PICK1-recruited PKCα-mediated phosphorylation of CKAMP44 alters AMPAR surface expression during synaptic plasticity events and whether it occurs at endosomal compartments.

The study of auxiliary AMPAR subunits is a rapidly evolving area of AMPAR trafficking research. Although much insight has been gained on understanding the ability of TARPs to alter AMPAR trafficking and channel properties, much more work needs to be done to elucidate how the lesser studied AMPAR auxiliary subunits regulate these processes.

### Linking Membrane Budding to AMPARs

The vesicular transport hypothesis states that intracellular trafficking of cargo between intracellular compartments in mammalian cells occurs via the encapsulation of cargo proteins, such as AMPARs, into small vesicles that bud from a donor compartment and fuse with an acceptor compartment (Bonifacino and Glick, 2004). Proteins involved in the early stages of membrane budding at plasma membrane and endosomal compartments form complexes of vesicle coating proteins, such as clathrin, APs and additional accessory proteins to link cargo to the nascent bud and coordinate subsequent trafficking (Lee and Goldberg, 2010).

Endocytosis from the plasma membrane and sorting into LEs often involves protein interactions with regions on the cargo proteins that are rich in lysine and arginine residues (Heo et al., 2006; Yeung et al., 2008), which are present in the cytoplasmic tails of AMPARs. This KR-rich region of AMPARs shows high homology across all its subunits, and is the region on GluA2 that binds the endocytic AP complex AP-2 (Lee et al., 2002). In addition, AMPAR sorting into LEs and subsequent lysosomal degradation in response to LTD induction is mediated by the KR-rich region of GluA2 (Lee et al., 2004). While classical AP complexes for lysosomal sorting (e.g., AP-3) have not been shown to bind to this site, the GluA2 interacting proteins cortactin and NSF play an important role. NSF can disrupt PICK1-GluA2 interactions, and hence regulate lysosomal trafficking of AMPARs (Hanley et al., 2002; Lee et al., 2004; Koszegi et al., 2017). It has recently been shown that the cortactin-AMPAR interaction maintains surface and total levels of GluA2/3-containing AMPAR by facilitating their recycling (Parkinson et al., 2018). NMDAR stimulation disrupts the interaction, resulting in the trafficking of AMPARs to lysosomes and their consequent degradation.

Nevertheless, a mechanism that does involve AP-3 has been identified, whereby the μ3A subunit of AP-3 interacts with the C-terminal tail of TARPγ2 in a phosphorylation-dependent manner to promote the late endosomal/lysosomal trafficking of AMPARs during NMDAR-dependent LTD (Matsuda et al., 2013). However, a recent study suggests that μ3A, functioning independently of the AP-3 complex, actually promotes the recycling of AMPARs to the plasma membrane during homeostatic scaling-up (Steinmetz et al., 2016). This mechanism also requires the GluA2-binding scaffolding protein GRIP.

Epidermal growth factor receptor substrate 15 (eps15) is an endocytic accessory protein that binds to AP-2 and contains a ubiquitin interacting motif and three Eps15 homology (EH) domains, which are widely present in endocytic accessory proteins (Benmerah et al., 1998; Confalonieri and Di Fiore, 2002). Eps15 has been shown to bind to ubiquitinated GluA1-containing AMPARs, facilitate their clathrin-dependent internalization and subsequent trafficking to lysosomes following neuronal stimulation (Lin and Man, 2014).

Sorting receptors are transmembrane proteins that couple cargo proteins to vesicle coats at the endoplasmic reticulum for efficient transport to the Golgi (Dancourt and Barlowe, 2010). The sorting receptor SorCS1, localizes to neuronal EEs and REs, interacts with AMPARs and regulates their basal surface expression (Savas et al., 2015). A related sorting receptor, SorCS3, interacts with PSD95 and PICK1 and has been proposed to promote synaptic efficacy through retaining surface AMPAR expression (Christiansen et al., 2017).

Retromer is a heteromeric complex of 5 proteins that was originally identified as a crucial mediator of retrograde trafficking, but is now emerging as an important regulator of recycling and lysosomal pathways (Burd and Cullen, 2014). It is a large complex comprising vacuolar protein sorting (VPS) proteins that constitute the “cargo selective complex,” which recognize and bind to cargo, and sorting nexins (SNXs), which link the cargo to the budding vesicle. AMPARs have recently been shown to utilize the retromer complex for local delivery from dendritic endosomes to synapses during basal trafficking (Choy et al., 2014; Tian et al., 2015) and activity-dependent trafficking in response to LTP induction (Temkin et al., 2017). SNXs contain a PX domain that interacts primarily with PI(3)P lipids, which are typically present on endosomal compartments (Gillooly et al., 2000; Teasdale and Collins, 2012). SNX27 also contains a PDZ domain and associates with GluA1 to promote AMPAR recycling during LTP and under basal conditions (Hussain et al., 2014; Loo et al., 2014).

The proteins described in this section link the clustered and primed native AMPAR complexes to particular trafficking membranes via coating, adaptor and accessory proteins that begin the membrane deformation process. Although some studies have identified proteins that sort AMPARs into recycling tubules or late endosomal compartments, most of the known machinery that regulates mammalian receptor sorting, for example ESCRT proteins, coat protein complex (COP) I and II, and other APs such as AP-4 and AP-5, have yet to be investigated in the specific context of AMPAR trafficking.

### Membrane Lipid Composition and Curvature

Endosomal membranes exhibit varying degrees of curvature and are marked by a unique complement of lipids, the most defining of which are the phosphoinositides (Ueda, 2014). AMPAR-associated proteins that differentially recognize phospholipids can target AMPARs to specific membranes and thus play crucial roles in coordinating the molecular events at the intersection between one membrane and the next. Furthermore, the regulation of phosphoinositide membrane identity via lipid de/phosphorylation is an important aspect of AMPAR trafficking. For example, the phosphoinositide-3-kinase (PI3K)-mediated phosphorylation of phosphatidylinositol 4,5-bisphosphate (PI(4,5)P_2_) to phosphatidylinositol (3,4,5)-trisphosphate (PIP_3_) and its reciprocal dephosphorylation by PTEN regulate the local accumulation of PIP_3_ at AMPAR-containing intracellular membrane compartments. Homotypic fusion of these intracellular AMPAR pools and PIP_3_-rich plasma membrane domains result in AMPAR synaptic insertion during LTP (Man et al., 2003; Arendt et al., 2010; Moult et al., 2010; Chan et al., 2011). Conversely, PTEN activity has been shown to depress AMPA receptor-mediated synaptic transmission and is required for NMDAR-dependent LTD, which is consistent with a loss of PIP_3_-containing plasma membrane identity to promote AMPAR synaptic removal (Jurado et al., 2010). PI(4,5)P_2_ is enriched at the plasma membrane and at intracellular membranes often associated with degradative pathways (Tan et al., 2015). The lipid phosphatase synaptojanin dephosphorylates PI(4,5)P_2_, so that endocytic vesicles can lose their plasma membrane identity, uncoat and traffic to the next intracellular compartment (Cremona et al., 1999). Although synaptojanin activity is required for AMPAR endocytosis (Gong and De Camilli, 2008), more recent work suggests that synaptojanin could also regulate the sorting of receptors at endosomal compartments. These studies demonstrate that disrupting synaptojanin function results in an intracellular accumulation of endosomal structures, suggesting that synaptojanin facilitates the maturation of endocytic vesicle membrane identity to allow the correct onward trafficking of cargo (Cossec et al., 2012; George et al., 2014).

PIP_3_ in endosomal membranes may also be phosphorylated by PIKfyve to generate PI(3,5)P_2_, which is thought to facilitate basal Rab11-mediated AMPAR synaptic delivery (Seebohm et al., 2012). However, PIKfyve has also been shown to drive synaptic depression by phosphorylating PI(3)P at plasma membranes to generate a more endosomal PI(3,5)P_2_ identity and induce AMPAR internalization (Zhang et al., 2012; McCartney et al., 2014). MTMR2 is a 3-phosphatase specific for the phosphoinositides PI(3)P and PI(3,5)P_2_ (Nicot and Laporte, 2008) and in neurons, acute knockdown of this phosphatase has been shown to enhance GluA2 trafficking to lysosomal compartments (Lee et al., 2010). This is proposed to occur through MTMR2-PSD95 interactions, which localize MTMR2 to excitatory synapses, where it prevents endosomal entry and subsequent lysosomal degradation.

PICK1 contains a BAR domain and a PDZ domain and regulates AMPAR trafficking during basal conditions and in response to LTD induction (Terashima et al., 2004; Steinberg et al., 2006; Terashima et al., 2008). PICK1 binds directly to GluA2, and is involved in AMPAR endocytosis (Fiuza et al., 2017) and endosomal sorting. At endosomal membranes, PICK1 restricts AMPAR recycling to the plasma membrane and may be involved in trafficking to lysosomes (Lin and Huganir, 2007; Citri et al., 2010; Koszegi et al., 2017). It has been suggested that the PICK1 BAR domain preferentially binds mono-phosphoinositides typically associated with endosomal compartments (Jin et al., 2006; Ueda, 2014), while the PDZ domain associates with a number of phospholipids, including PI(4,5)P_2_ (Pan et al., 2007).

Another BAR-domain containing protein that has been implicated in AMPAR trafficking is PKC and casein kinase II substrate in neurons (PACSIN, also known as Syndapin), which forms a complex with PICK1 and AMPARs to facilitate the activity-dependent internalization of AMPARs for the expression of cerebellar LTD (Anggono et al., 2013). More detailed investigation into the role of PACSIN1 in AMPAR trafficking suggested that PACSIN plays dual roles in AMPAR trafficking; the PICK1-PACSIN1 interaction is essential in activity-dependent recycling of AMPARs, and the SH3 and F-BAR interactions of PACSIN1 with as yet unidentified partners are important in AMPAR endocytosis (Widagdo et al., 2016).

## AMPAR Endosomal Sorting in Disease

There is significant overlap between endo-lysosomal, autophagosomal and ubiquitin-mediated degradation (Korolchuk et al., 2010; Cohen-Kaplan et al., 2016) and the dysregulation of these systems has been consistently observed in neurodegenerative diseases (Nedelsky et al., 2008; Lee et al., 2013). Indeed, endosomal dysfunction is an early indicator for a number of neurodegenerative diseases, such as Alzheimer’s disease (AD) and Parkinson’s disease (PD; Schreij et al., 2016), Niemann-Pick type C1 (D’Arcangelo et al., 2011; Rabenstein et al., 2017), and other neuropathologies, such as ischemia (Yuan et al., 2018). Crucially, these endosomal deficits result in aberrant lysosomal targeting of crucial synaptic proteins, such as AMPARs, and is thought to underlie the impairments in learning and memory seen in these pathologies. Thus, strategies that promote correct endo/lysosomal fusion, maturation and trafficking of AMPARs are gaining momentum as viable clinical targets in neuropathologies that exhibit endosomal dysfunction (Friedman et al., 2015; Wen et al., 2017). Furthermore, the distinct spatio-temporal differences in the auxiliary subunit composition of native AMPAR complexes throughout the brain (Schwenk et al., 2014), shows promise for the development of targeted therapies for various neuropathologies. Indeed, studies are beginning to develop drugs that preferentially target AMPAR complexes that include specific auxiliary subunits such as stargazin (Azumaya et al., 2017).

Brain ischemia occurs when the blood supply to the brain is interrupted via stroke or cardiac arrest, which causes neuronal depolarization, excessive glutamate release, AMPAR overexcitation and a sustained raise in intracellular Ca^2+^ (Arundine and Tymianski, 2004). This excitotoxic Ca^2+^ signaling is caused in part by a reduction in synaptic GluA2-containing AMPARs, and the subsequent expression of GluA2-lacking Ca^2+^-permeable AMPARs at synapses. This switch is a multi-stage process involving GluA2-dependent endocytosis from the plasma membrane, and also the aberrant trafficking of GluA2-containing AMPARs to lysosomes, where they are degraded (Liu et al., 2006; Blanco-Suarez and Hanley, 2014; Koszegi et al., 2017). A persistent reduction in GluA2 expression after ischemia is maintained by a reduction in mRNA levels by regulation at the level of transcription (Pellegrini-Giampietro et al., 1992; Gorter et al., 1997). The ectopic presence of autolysosomes is increasingly observed from 1 h to 12 h after permanent middle cerebral artery occlusion (experimental stroke; Wen et al., 2008). Therefore, clearing ectopic lysosomes and preventing improper degradation of GluA2-containing AMPARs have potential as therapeutic strategies for brain ischemia.

AD is characterized by an abnormal intracellular accumulation of neurofibrillary tangles of hyper-phosphorylated tau (Iqbal and Grundke-Iqbal, 2008) and extracellular amyloid-β (Aβ) plaques (Selkoe and Hardy, 2016). In the early stages of AD pathology, Rab5 and Rab7 are upregulated (Ginsberg et al., 2010), which is proposed to precede and exacerbate the accumulation of Aβ protein aggregates (Takahashi et al., 2002, 2004; Capetillo-Zarate et al., 2011). Moreover, Aβ42, the amyloid peptide that is most prone to aggregation, accumulates in LEs and impairs endosomal sorting of neuronal cargo to be degraded, since they cannot translocate from the outer membrane to the inner membrane of MVBs in LEs (Cataldo et al., 2000, 2003; Almeida et al., 2006; Burns and Rebeck, 2010; Jiang et al., 2010; Choi et al., 2013). Impaired endosomal sorting is thought to decrease the surface expression of AMPARs and cause the cognitive defects seen in AD (Almeida et al., 2005; Chang et al., 2006; Hsieh et al., 2006; Ting et al., 2007). The detailed molecular mechanisms of this AMPAR loss remain elusive, but a recent study has shown that Aβ increases Nedd4-1-mediated AMPAR ubiquitination, and knockdown of Nedd4-1 can rescue Aβ-induced synaptic deficits (Alfonso et al., 2014; Rodrigues et al., 2016). The aberrant presence of GluA2-lacking, CP-AMPARs, which promotes excessive calcium signaling (LaFerla, 2002; O’Hare Doig et al., 2016) may hyper-phosphorylate tau and augment the formation of toxic Aβ oligomers (Mattson et al., 1993; Pierrot et al., 2006). Interestingly, Aβ selectively decreases GluA2-containing AMPARs through PKC-phosphorylation of serine 880 (Liu et al., 2010; Guntupalli et al., 2016) and causes a rapid synaptic insertion of CP-AMPARs (Whitcomb et al., 2015). Furthermore, GluA3 subunits are most readily associated with GluA2 subunits in the mature hippocampus (Wenthold et al., 1996; Lu et al., 2009) and mice lacking GluA3-containing AMPARs are protected against Aβ-induced synaptic deficits, spine loss and memory impairment (Reinders et al., 2016). Therefore, it may be the case that GluA3 subunits are predominantly responsible for AMPAR lysosomal sorting, which is supported by earlier studies (Lee et al., 2004). Preventing GluA2-containing AMPAR lysosomal sorting may hold potential for therapeutic intervention in AD, and targeting GluA3 could be an effective approach to prevent further loss of synaptic GluA2/3 heteromers.

Intraneuronal proteinaceous inclusions, termed Lewy bodies (LBs) that are enriched in α-synuclein are observed in PD (Dickson, 2012). Similar to the etiology of AD, these α-synuclein aggregations are thought to disrupt intracellular trafficking pathways at late endosomal and lysosomal compartments (Outeiro and Lindquist, 2003; Mazzulli et al., 2011; Volpicelli-Daley et al., 2014). Indeed, α-synuclein-induced disruption of the ESCRT-III complex results in decreased MVB formation, intracellular α-synuclein aggregation and its consequent exocytosis, which propagates the toxic effects to other neighboring cells (Spencer et al., 2015). Again, enhancing late endosomal function through Rab7 activation has been shown to clear pathological α-synuclein aggregates (Dinter et al., 2016), and AMPARs have been identified as important dysregulated trafficking cargo. For example, neurons expressing genetic mutations that are present in familial PD significantly reduce AMPAR surface expression (Cortese et al., 2016). Moreover, retromer dysfunction has also been heavily implicated in the improper trafficking of AMPARs from endosomes to the plasma membrane in AD (Muhammad et al., 2008; Lane et al., 2012), PD (Munsie et al., 2015) and other neurodegenerative conditions (Damseh et al., 2015). Moreover, extracellular α-synuclein increases synaptic CP-AMPAR expression (Diógenes et al., 2012) and so targeting GluA2-containing AMPARs and their strict control of intracellular Ca^2+^ is again of significant clinical importance.

## Concluding Remarks

The process of endosomal sorting is extremely complex, especially in the context of the extended and polarized morphology of central neurons. Moreover, the system needs to be highly dynamic and respond to different types of synaptic stimulation, and to respond in a local, synapse-specific manner to change the receptor complement of individual synapses. Despite this complexity, significant progress has been made in elucidating the relevant mechanisms. An additional challenge is the interconnected nature of endosomal and vesicular membranes, which leads to experimental difficulties in precisely defining roles for regulatory proteins at specific intracellular compartments. However, future work employing more spatially resolved imaging techniques and better targeted molecular tools in neuronal systems will more completely define the roles of AMPAR trafficking proteins in endosomal sorting.

## Author Contributions

GP wrote the original draft of the manuscript. JH edited the manuscript and completed the final version.

## Conflict of Interest Statement

The authors declare that the research was conducted in the absence of any commercial or financial relationships that could be construed as a potential conflict of interest.
